# Effect of Gallic acid and Myricetin on ovarian cancer models: a possible alternative antitumoral treatment

**DOI:** 10.1186/s12906-020-02900-z

**Published:** 2020-04-10

**Authors:** Luis Varela-Rodríguez, Blanca Sánchez-Ramírez, Verónica Ivonne Hernández-Ramírez, Hugo Varela-Rodríguez, Rodrigo Daniel Castellanos-Mijangos, Carmen González-Horta, Bibiana Chávez-Munguía, Patricia Talamás-Rohana

**Affiliations:** 1 Departamento de Infectómica y Patogénesis Molecular, CINVESTAV-IPN. Ave. Instituto Politécnico Nacional No. 2508, Col. San Pedro Zacatenco, C.P. 07360 Mexico City, Mexico; 2 Facultad de Ciencias Químicas, Universidad Autónoma de Chihuahua, Circuito 1, Nuevo Campus Universitario, C.P. 31125 Chihuahua, Chih Mexico; 3 Laboratorio de Complejidad Molecular y Desarrollo, Unidad de Genómica Avanzada, CINVESTAV-IPN, Libramiento Norte Carretera Irapuato-León Km. 9.6, C.P, 36824 Irapuato, Gto Mexico; 4 Centro Médico ISSEMyM “Arturo Montiel Rojas”, Av. Baja Velocidad No. 284, Carretera México-Toluca Km 57.5, Col. San Jerónimo Chicahualco, C.P. 52170 Metepec, Edo. Mex Mexico

**Keywords:** Antineoplastic activity, *Nu/Nu* mice, OVCAR-3, Peritumoral route, SKOV-3, Toxicity, Xenotransplanted mice

## Abstract

**Background:**

Ovarian cancer is the leading cause of mortality among malignant gynecological tumors. Surgical resection and chemotherapy with intravenous platinum/taxanes drugs are the treatments of choice, with little effectiveness in later stages and severe toxicological effects. Therefore, this study aimed to evaluate the antineoplastic activity of gallic acid (GA) and myricetin (Myr) administrated peritumorally in *Nu/Nu* mice xenotransplanted with SKOV-3 cells.

**Methods:**

Biological activity of GA and MYR was evaluated in SKOV-3 and OVCAR-3 cells (ovarian adenocarcinomas) by confocal/transmission electron microscopy, PI-flow cytometry, H_2_-DCF-DA stain, MTT, and Annexin V/PI assays. Molecular targets of compounds were determined with ACD/I-Labs and SEA. Antineoplastic activity was performed in SKOV-3 cells subcutaneously xenotransplanted into female *Nu/Nu* mice treated peritumorally with 50 mg/kg of each compound (2 alternate days/week) for 28 days. Controls used were paclitaxel (5 mg/kg) and 20 μL of vehicle (0.5% DMSO in 1X PBS). Tumor lesions, organs and sera were evaluated with NMR, USG, histopathological, and paraclinical studies.

**Results:**

In vitro studies showed a decrease of cell viability with GA and Myr in SKOV-3 (50 and 166 μg/mL) and OVCAR-3 (43 and 94 μg/mL) cells respectively, as well as morphological changes, cell cycle arrest, and apoptosis induction due to ROS generation (*p* ≤ 0.05, ANOVA). In silico studies suggest that GA and MYR could interact with carbonic anhydrase IX and PI3K, respectively. In vivo studies revealed inhibitory effects on tumor lesions development with GA and MYR up to 50% (*p* ≤ 0.05, ANOVA), with decreased vascularity, necrotic/fibrotic areas, neoplastic stroma retraction and apoptosis. However, toxicological effects were observed with GA treatment, such as leukocyte infiltrate and hepatic parenchyma loss, hypertransaminasemia (ALT: 150.7 ± 25.60 U/L), and hypoazotemia (urea: 33.4 ± 7.4 mg/dL), due to the development of chronic hepatitis (*p* ≤ 0.05, ANOVA).

**Conclusion:**

GA and Myr (50 mg/kg) administered by peritumoral route, inhibit ovarian tumor lesions development in rodents with some toxicological effects. Additional studies will be necessary to find the appropriate therapeutic dose for GA. Therefore, GA and Myr could be considered as a starting point for the development of novel anticancer agents.

## Background

Ovarian cancer is the leading cause of death from malignant gynecological tumors, and the fourth most common cause of cancer death in women [1]. Currently, there is no effective screening program, and there are few specific symptoms/signs in the disease. The main histological subtypes are epithelial (70%), germinal (20%) and stromal (10%) [1]. The main treatment for this disease is surgical resection followed by chemotherapy with platinum/taxanes drugs by the intravenous pathway [2, 3]. However, these schemes have low effectiveness in late stages of the disease, and in some cases produce severe toxicological effects [4]. For these reasons, research of new routes of administration and new therapeutic candidates for this disease is necessary. Studies done by Wright et al. (2015), demonstrated that the intraperitoneal (i.p.) administration of chemotherapy in randomized trials with ovarian cancer patients, increased the survival of patients by 16 months, compared with intravenous chemotherapy alone; whereby, the administration of i.p. chemotherapy in the clinical practice could be an important strategy to treat this pathology [5].

Some compounds found in plants have shown anti-cancer activity and thus are used for the treatment of this pathology [6]; such is the case for paclitaxel obtained from *Taxus brevifolia* [7], vincristine from *Catharanthus roseus* [8] and curcumin from *Curcuma longa* [9], which show different mechanisms of action against cancer cells.

Polyphenolic compounds have attracted attention in recent decades for their beneficial effects on health, by preventing or/and combating diseases associated with oxidative stress such as cardiovascular/neurodegenerative pathologies and cancer [10–13]. The primary mechanism of action of these compounds is their anti/pro-oxidant effect [14–16]. Some examples of polyphenols with anti cancer effect are flavonoids such as quercetin, kaempferol, fisetin, myricetin (Myr), and phenolic acids such as gallic acid (GA), protocatechuic acid and rosmarinic acid [6, 8, 10, 17]. Recent studies have demonstrated that Myr and GA particularly, could have interesting applications in the treatment of cancer [18, 19].

Myr (3,5,7-trihydroxy-2-(3,4,5-trihydroxyphenyl)chromen-4-one) is a flavonoid present in some plants families such as *Myricaceae, Anacardiaceae, Polygonaceae, Pinaceae,* and *Primulaceae*. Usually, Myr is found as a glycosylated variant denominated myricitrin (myricetin 3-*O*-rhamnoside) [20, 21]. Previous works have demonstrated the beneficial effects of Myr against different types of cancer, through the inhibition of protein kinases in distinct intracellular signaling pathways such as PI3K-PKB/Akt/mTOR, MEK1, Fyn and JAK1-STAT3, among others [22–24]. While, GA (3,4,5-trihydroxy benzoic acid) is widely distributed in plants as phenolic acid polymers (condensed tannins); GA presents anti-cancer activity through several pharmacological and biochemical pathways, such as: ATM/Chk2/p53 activation (cell cycle arrest/apoptosis induction), H2A.X/ribonucleotide reductase inhibition (DNA synthesis inhibition by free radical scavengers and alteration in dNTP balance), COX-2/NF-kB inhibition (anti-inflammatory effect) and GSH depletion (anti-oxidant effect) [25, 26].

Thus, GA and Myr may be attractive candidates for ovarian cancer treatment, because recent studies showed that these compounds present activity against stomach, colon, and prostate cancers [22–26]. Therefore, this study aimed to evaluate the antineoplastic activity of GA and Myr, first in vitro against human ovarian cancer cell lines (SKOV-3 and OVCAR-3), and then in vivo by peritumoral administration in SKOV-3 cells xenotransplanted in *Nu/Nu* mice.

## Methods

### Material and compounds studied

Compounds evaluated in this study were GA (G7384) (50 μg/mL in cells or 50 mg/kg of body weight in mice) and Myr (M6760) (166 μg/mL in cells or 50 mg/kg of body weight in mice) from Sigma-Aldrich© Chemical Co. (St. Louis, Missouri, EE.UU.) with a ≥ 96% purity (HPLC-grade). Paclitaxel (5 μg/mL in cells or 5 mg/kg body weight in mice) (Sigma®), a drug used for ovarian cancer treatment, and vehicle (0.5% DMSO in 1X PBS, *v/v*) were used as positive and negative controls, respectively. Additional use of equipment and reagents are indicated in the text.

### Cell culture protocol

Cell lines used for this study were: SKOV-3 (HTB-77™, ATCC®) and OVCAR-3 (HTB-161™, ATCC®) from ovarian adenocarcinomas, and transformed/non-tumorigenic BEAS-2B (CRL-9609™, ATCC®) from lung/bronchus human epithelium. Cell monolayers were maintained according to the supplier’s instructions at 37 °C, 95% humidity, and 5% CO_2_. Cells were harvested using 1X trypsin-EDTA solution (Sigma®), and the cell density at collection time was determined by Trypan blue (0.4%, Sigma®) stain.

### Cell viability by MTT assay

Cells (2 × 10^4^ per well) were placed in 96-well flat-bottom plates (Corning®) with 200 μL of supplemented medium (Gibco™) and incubated for 24 h. Cells were treated with experimental compounds (concentrations from 10 to 200 μg/mL) for 24 h, and 20 μL of MTT (5 mg/mL in 1X PBS, Sigma®) were added 4 h before the end of the incubation time. Vehicle (0.5% DMSO in 1X PBS, *v/v*) was used as a negative control, and Paclitaxel (5 μg/mL, Sigma®), a drug used for the treatment of ovarian cancer, was used as a positive control. Next, the culture medium was removed, and the formazan produced by the cells was measured at 590 nm in a microplate reader (Model 680, Bio-Rad®) [27, 28]. The cell viability and half-maximal inhibitory concentration (IC_50_) were calculated as follows [27]: *% Cell viability = (Abs*_*sample*_*/ Abs*_*control*_) * 100 and regression analysis (percentage survival vs log concentration), respectively.

### Cell morphology evaluation by immunofluorescence

Cells (3 × 10^4^ per well) were placed in Lab-Tek™ chamber slides (Thermoscientific®) with 400 μL of supplemented medium (Gibco™) for 24 h. Adherent cells were treated with IC_50_ of samples and controls for 24 h. Then, the culture medium was removed, and cells were fixed with 2% paraformaldehyde (Sigma®) for 30 min at 37 °C. Next, cells were permeabilized with 0.2% Triton-X100 (Sigma®) for 15 min and blocked with 10% FBS (Gibco™) for 1 h at 37 °C. Microtubules were labeled with an *α*/*β*-tubulin primary polyclonal antibody (55 kDa, rabbit) (1:200 μL, *v/v*) (Cell Signaling©) for 12 h at 4 °C and a secondary donkey/anti-rabbit IgG-FITC antibody (1:100 μL, *v/v*) (Jackson Immuno Research©) for 1 h at 37 °C. Actin microfilaments were labeled with Rhodamine-Phalloidin (2:100 μL, *v/v*) (ThermoFisher®) for 30 min at 25 °C. Finally, preparations were mounted with VectaShield®/DAPI (Vector Laboratories®) and observed in a confocal microscope (LSM 700, Zeiss®) at 40X and analyzed with the ZEN® 2011 software (Version 1.0, Zeiss®) [29].

### Ultrastructural morphology by transmission electron microscopy (TEM)

Cells (5 × 10^5^ per well) were placed in 6-well flat-bottom plates (Corning®) with 2 mL of supplemented medium (Gibco™) for 24 h. Adherent cells were treated with IC_50_ of samples and controls for 24 h. Then, the culture medium was removed, and cells were fixed with 2.5% glutaraldehyde (Sigma®) for 24 h at 25 °C. Next, cells were washed with 0.1 M sodium cacodylate (Sigma®) (pH 7.2), and post-fixed with 1% osmium tetraoxide (Sigma®) for 1 h at 25 °C. Subsequently, cells were dehydrated with EtOH: propylene oxide (C_3_H_6_O, Sigma®) (50, 70, 90, 100% EtOH and 100% C_3_H_6_O, *v/v*) for 10 min at 4 °C. The inclusion of the cells was done with Poly/Bed® 812 epoxy resin (Polysciences®) at 60 °C for 24 h. Finally, ultrathin sections (60 nm thickness) were obtained with an ultramicrotome (Porter-Blum MT-1, Sorvall®). The slices were contrasted with 2% uranyl acetate (Polysciences®) for 20 min, and 0.2% lead citrate (Polysciences®) for 5 min. The observation of preparations was carried out in a TEM (JEM-1100, Jeol™) [30].

### Cell cycle analysis by flow cytometry

Cells (5 × 10^5^ per well) were placed in 6-well flat-bottom plates (Corning®) with 2 mL of supplemented medium (Gibco™) for 24 h. Adherent cells were treated with IC_50_ of samples and controls (20 μg/mL mitomycin, Sigma®) for 24 h. Then, the culture medium was removed, and cells were detached by trypsinization, fixed and permeabilized with 50% EtOH (J.T.Baker®) at − 20 °C for 12 h. Finally, cells were washed and pelleted by centrifugation (2000 rpm, 10 min, 4 °C) to add 400 μL 1X PBS, 10 μL RNAsa A (10 mg/mL, Sigma®) and 20 μL propidium iodide (1 mg/mL PI, Invitrogen®) during 1 h at 37 °C. The DNA content in each cell cycle stage was analyzed in a BD FACS-Calibur™ (Becton Dickinson®) and data processed by the ModFit LT software (version 5.0, Verity Software House®) [28, 31].

### Cell death determination by AnV/PI

Cells (2 × 10^4^ per well) were placed in 96-well flat-bottom black plate (Corning®) with 200 μL of supplemented medium (Gibco™) for 24 h. Adherent cells were treated with IC_50_ of samples and controls for 24 h. Then, the culture medium was removed and 200 μL of 1X binding buffer (10 mM HEPES, 140 mM NaCl and 2.5 mM CaCl_2_, at pH 7.4), 2 μL of AnV-FITC and PI (250 μg/mL) (BioVision™) were added for 15 min at 37 °C. Finally, fluorescence was quantified at λ_ex/em_ 485/538 nm (AnV-FITC) and λ_ex/em_ 538/620 nm (PI) in FluorosKan® Ascen FL (Termoscientific®). The assay was corroborated by confocal microscopy (LSM 700, Zeiss®) at 40X. Cells were identified as: viable (AnV-/PI-), necrotic (AnV-/PI+), or on early/late apoptosis (AnV+/PI- or AnV+/PI+), respectively.

### ROS-intracellular quantification by H_2_-DCF-DA

Cells (2 × 10^4^ per well) were seeded in 96-well flat-bottom black plate (Corning®) with 200 μL of supplemented medium (Gibco™) for 24 h. Adherent cells were treated with IC_50_ of samples and controls (0.3% H_2_O_2_, Sigma®) for 24 h. Then, 25 μM H_2_-DCF-DA (Sigma®) was added for 15 min at 37 °C. Finally, fluorescence was quantified at λ_ex/em_ 488/529 nm in FluorosKan® Ascen FL (Termoscientific®). The assay was corroborated by confocal microscopy (LSM 700, Zeiss®) at 40X.

### Antineoplastic activity of GA and Myr

**Fig. 1:**
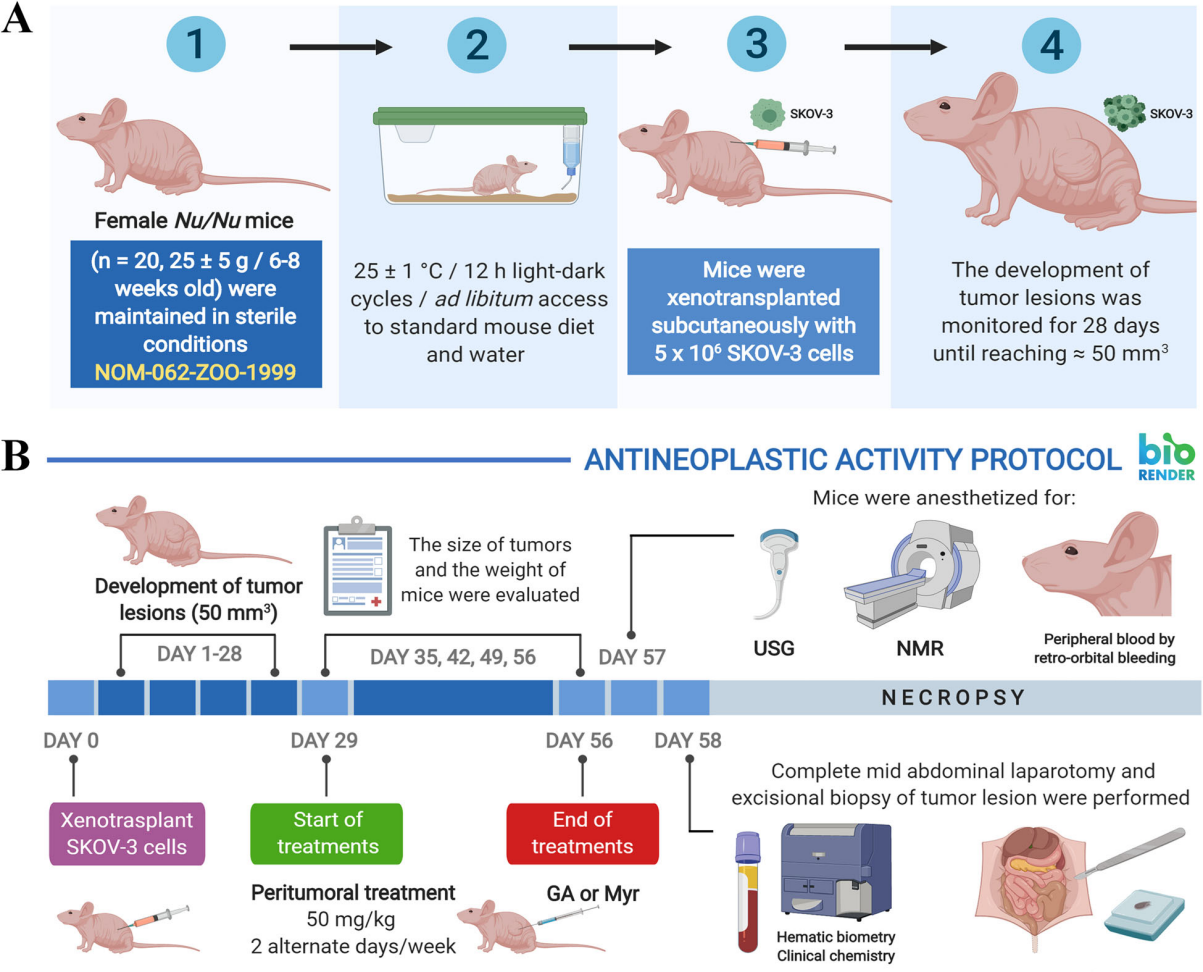
Timeline of the antineoplastic protocol implemented for GA and Myr. The antineoplastic activity of GA and Myr was determined in SKOV-3 cells xenotransplanted into female *Nu/Nu* mice as described in methods, according to the protocol proposed by Zou et al., 2007 [33] and the Official Mexican Regulations [34]. GA, gallic acid; Myr, myricetin; NMR, nuclear magnetic resonance; USG, ultrasonography

Athymic nude mice (*Mus musculus Nu/Nu,* Crl:NU*-Foxn1*^*nu*^ Immunodeficient Outbred) were acquired from Charles River Laboratories Inc., US, in 2017 by CINVESTAV-IPN. Supplier health reports indicated that the mice were free of known viral, bacterial and parasitic pathogens. This animal model has a simple and spontaneous mutation that generate abnormal hair growth and defective development of the thymic epithelium. Whereby, the mice phenotypically lack hair (albino background), and present a functional rudimentary thymus which produces a reduced number of mature T-cells, and thus, do not reject allogenic and xenotransplanted tissues. In addition, these mice have a normal complement system and B-lymphocytes dependent immune responses. Therefore, *Nu/Nu* mice are ideal for research of tumor biology, and anti-cancer therapies, among other general purposes [32]. For this study, adult homozygous female mice were selected with 25 ± 5 g body weight and 6–8 weeks old, maintained and reproduced in sterile conditions at 25 ± 1 °C, 50 ± 3% humidity, with 12 h light-dark cycles and ad libitum access to sterile standard mouse diet (LabDiet®) and sterile water, in a controlled room of the Animal Production and Experimentation Unit (UPEAL) from CINVESTAV-IPN (Fig. 1a-1). Mice were housed in autoclavable polycarbonate boxes (5 mice per box), with AISI lid and dimensions of 470 mm × 290 mm × 190 mm. All boxes contained sterile wood shavings as bedding. Additionally, the health state of animals and their adaptation to the new conditions of laboratory were evaluated for a period of 15 days, to decrease their stress and anxiety levels. This study was carried out according to the protocol proposed by Zou et al., 2007 [33] and the Official Mexican Regulations [34]. Also, the study was approved by the Ethics Committee from UPEAL (Protocol No. 0184–16) (Section: Ethics approval and consent to participate) and mice were not subjected to any previous experimental procedure or any additional treatment, before beginning the experimental procedures.

**Fig. 2:**
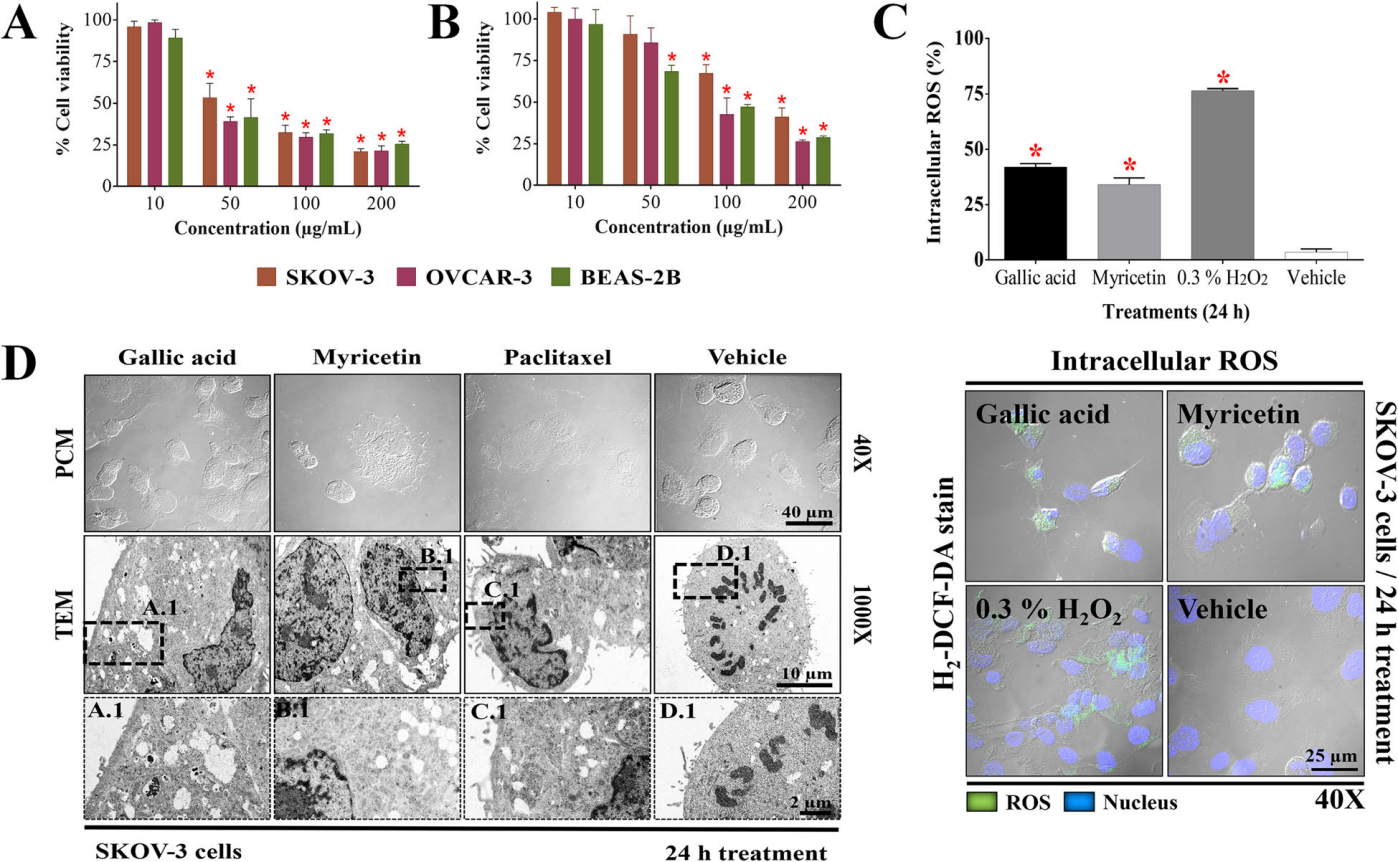
Biological activity of GA and Myr in ovarian cancer cells. The IC_50_ of GA (**a**) and Myr (**b**) in SKOV-3, OVCAR-3, and BEAS-2B cell lines was obtained with dose-response viability curves at 24 h by MTT assay. Intracellular production of ROS (**c**) and ultra-structural changes (**d**) in SKOV-3 cells were analyzed by H_2_-DCF-DA and TEM respectively, after 24 h of treatment with GA (50 μg/mL) and Myr (166 μg/mL). The boxes represent optical magnification made in TEM analysis for the corresponding treatments (A.1 GA, B.1 Myr, C.1 paclitaxel, D.1 vehicle). Results show the mean ± S.D. of three biological replicates (*n* = 3, in triplicates); *, *p* ≤ 0.05 vs the control group without treatment (0.5% DMSO in 1X PBS - 100% viability, ANOVA). Paclitaxel was used as a positive control at 5 μg/mL. ROS, reactive oxygen species; PCM, phase-contrast microscopy; TEM, transmission electron microscopy

**Fig. 3:**
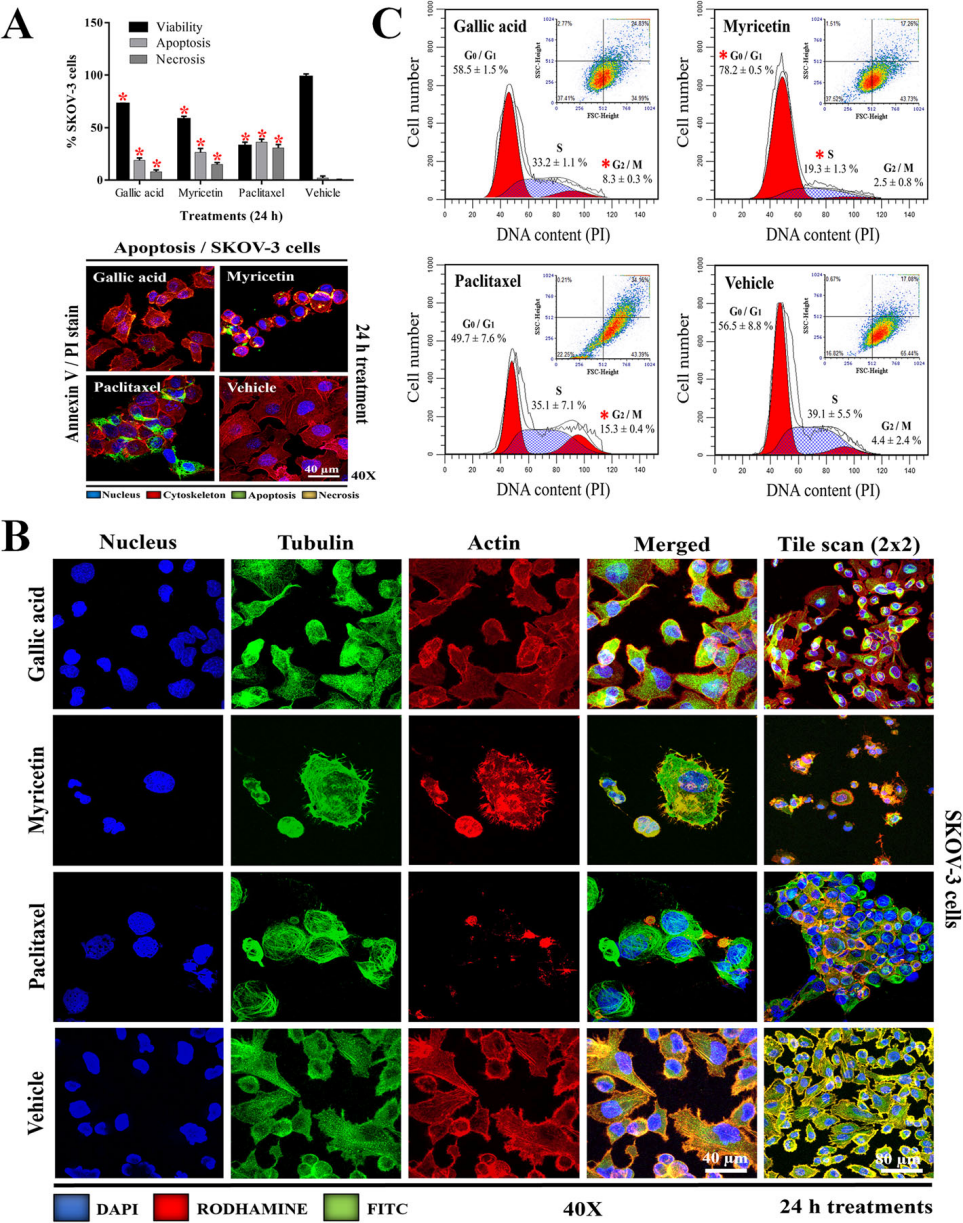
Cytological effects of GA and Myr in SKOV-3 cells. Apoptosis induction (**a**), morphological changes (**b**), and DNA content in different phases of the cell cycle (**c**) were determined by flow cytometry, after 24 h of treatment with GA (50 μg/mL) and Myr (166 μg/mL) and using: Annexin-V, immunofluorescence with *α*/*β*-tubulin antibody, and propidium iodide, respectively. Results show the mean ± S.D. of three biological replicates (*n* = 3, in triplicates); *, *p* ≤ 0.05 vs. the control group without treatment (0.5% DMSO in 1X PBS, ANOVA). Paclitaxel was used as a positive control at 5 μg/mL. PI, propidium iodide

**Fig. 4:**
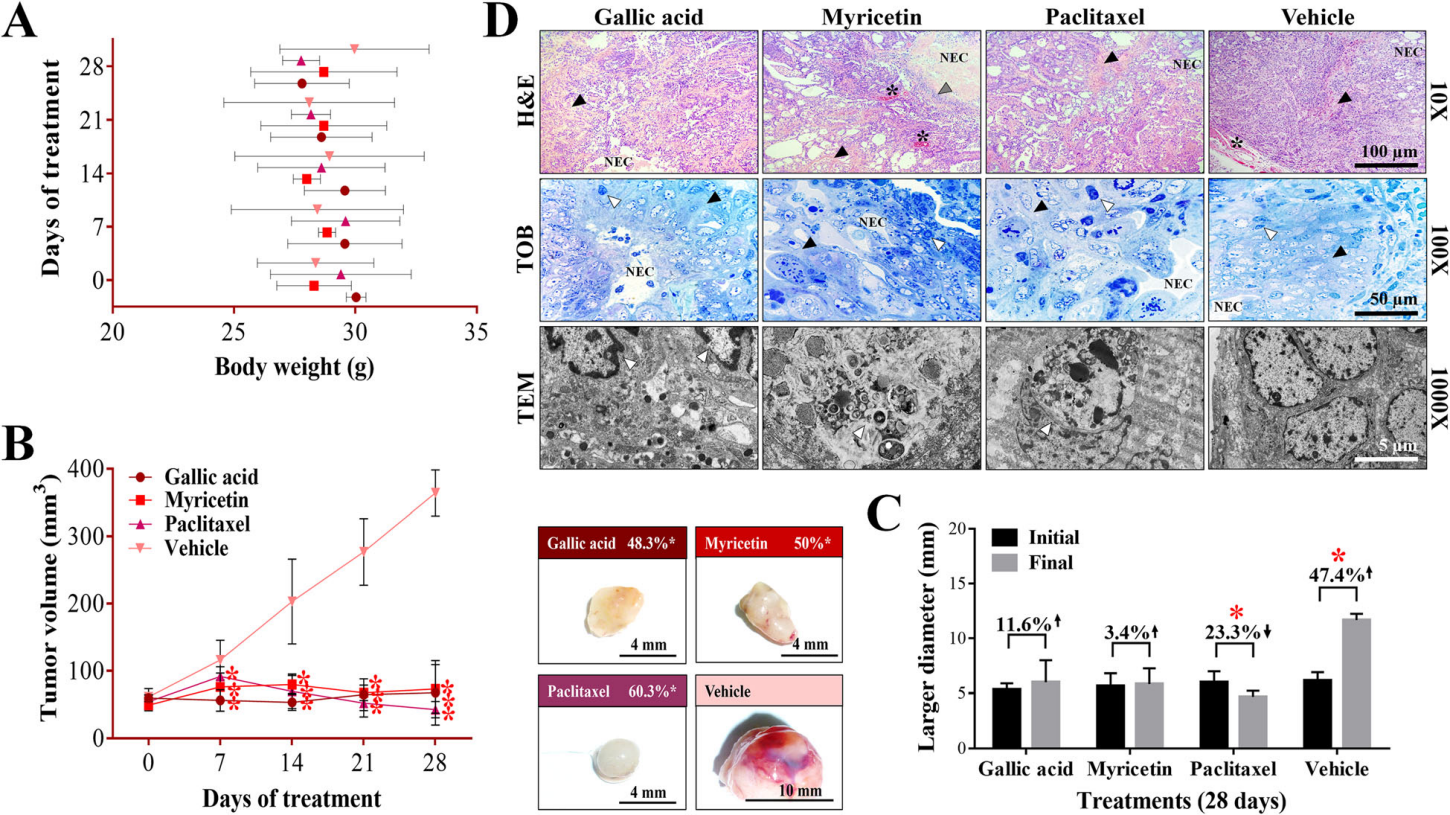
Antineoplastic activity of GA and Myr in mice xenotransplanted with ovarian cancer. The body weight of rodents was monitored with an electronic bascule for 28 days (**a**). The tumor volume was determined with a Vernier caliper in mice treated with GA and Myr for 4 weeks, with doses of 50 mg/kg in 2 alternate days per week by peritumoral route (Tumoral volume = [Larger diameter * (Shorter diameter)^*2*^*] / 2*). Additionally, morphological changes in tumor lesions were analyzed at the end of the treatments, and the % inhibition was calculated (**b**). Subsequently, the disease evolution was evaluated in each treatment with the larger diameter obtained in tumoral lesions at the beginning and final of the assay. Also, the % inhibition of tumoral volume was determined based on the final volume of each tumor after treatment concerning the final volume obtained by the control group (**c**). Differential histological patterns were observed in the tumor lesions by H&E / TOB stain and by TEM (**d**). Results show the mean ± S.D. of two biological replicates (*n* = 5); *, *p* ≤ 0.05 vs. the control group without treatment (20 μL of 0.5% DMSO in 1X PBS, ANOVA). Paclitaxel was used as a positive control at 5 mg/kg body weight, administered under the same conditions that experimental samples. The arrow’s direction indicates gain (↑) or loss (↓) of tumoral volume (**c**). The arrows and symbols indicate: fibrosis (black arrowhead), necrotic area (NEC), vascularization (*), leukocytic infiltrates (grey arrowhead), and apoptotic cells (white arrowhead) (**d**). H&E, hematoxylin and eosin; TOB, toluidine blue; TEM, transmission electron microscopy

Twenty mice were randomly divided into 4 groups (*n* = 5) (Fig. 1a-2) [35], and 5 × 10^6^ SKOV-3 cells in 100 μL of 1X PBS were subcutaneously xenotransplanted in mice’s right flanks (Fig. 1a-3). The development of tumor lesions was monitored during 28 days until growth reached ≈ 50 mm^3^ (Fig. 1a-4) [33]. Next, rodents were treated, peritumorally (p.t.), with 50 mg/kg of body weight of each compound (GA and Myr) or 5 mg/kg of body weight of controls (Paclitaxel and 0.5% DMSO in 1X PBS, *v/v*) in 2 alternate days per week, for 28 days (Fig. 1b). During the experiment, animals were evaluated at 24 h post-treatment to detect any signs discomfort/pain or toxicity [34, 35]. Additionally, the mice weight and tumoral lesions were determinated every week after the administration of treatments with an electronic bascule (CS200, Ohaus®) and a Vernier caliper (Truper®) respectively (Fig. 1b) [33–35]. The tumoral volume was calculated as [33]: *Tumoral volume (mm*^*3*^) = [Larger diameter * (Shorter diameter)^*2*^*] / 2*.

Throughout the studies, pentobarbital sodium (Pet’s Pharma®) was applied as anesthesic to mice at 0.1575 mg/250 μL 1X PBS (*v/v*), in the following cases and under the conditions specified below: i) for the xenotransplantation process (a single dose); ii) before the administration of the treatments (2 doses per animal/week, over 4 weeks); iii) at the end of the treatments for imaging and paraclinical studies (a single dose for each type of study). Finally and for euthanasia purposes, the same anesthetic was administered at a lethal concentration of 0.63 mg/100 μL 1X PBS (*v/v*) (single overdose). In all cases, the anesthetic was administered by i.p. injection, in mice placed supine position by grasping the nape and the skin fold between lower/middle back. The administration of analgesics was not necessary.

For the experimental procedures, female mice were selected because they are not territorial and do not show aggressive behavior (compared with males), a situation that could have interfered with the results of the study [35], and in the same way, the nature of the model used to study the antineoplastic activity of the compounds against ovarian cancer. In addition, the p.t. route was used to ensure direct contact of the drugs studied with tumor lesions and reduce their possible adverse side effects. Moreover, the p.t. route was selected for its similarity with the i.p. route used for the administration of drugs, during the chemotherapy of cancer patients [5].

### Imaging studies by nuclear magnetic resonance (NMR) and ultrasonography (USG)

For these studies, animals were anaesthetized as described above. NMR was performed with a Magnetom Symphony™ system, A Tim System 1.5 T eco (Siemens™), with knee antenna and without paramagnetic contrast. USG was performed on an ultrasound system with agile acoustic architecture (LogiQ™ P7, General Electric-Healthcare®) for real-time image with a multifrequency linear transducer for soft and vascular tissue (L6–12 RS, General Electric-Healthcare®) (4–13 Mhz band and 39 mm vision). After imaging studies, results were analyzed in the RadiAnt DICOM Viewer software (version 3.4, Medixant©), to perform the measurement and characterization of tumor lesions, as well as 3D reconstructions.

### Paraclinic and histopathologic studies

Peripheral blood samples were obtained by retro-orbital puncture in anesthetized animals using heparinized capillary tubes (Vitrex®) and collected in pediatric tubes with K_2_EDTA (BD Microtainer®) (Fig. 1b). Plasma was obtained by centrifugation (3500 rpm at 4 °C for 10 min). The hematic biometry and biochemical parameters were determined using a hematology autoanalyzer system (BC-2300, Mindray®) and automated medical system (Prestige® 24i, Tokyo Boeki®) respectively. Next, animals were euthanized by cervical fracture under anesthesia to perform a mid abdominal laparotomy for extraction of kidneys, heart, lungs, spleen, and liver (Fig. 1b). Additionally, an excisional biopsy was made at the tumor lesions site (Fig. 1b). Organs and tumors were rinsed with 1X PBS, weighted and adherent tissue removed. Subsequently, samples were fixed in 4% paraformaldehyde (Sigma®) and paraffin-embedded to obtain thick sections (5 μm thickness) with a rotatory microtome (RM2125 RTS, Leica®). Tissue slices were stained with hematoxylin-eosin (Merck®) or toluidine blue (TOB, Sigma®) and observed by optical microscopy (BX41, Olympus®). Finally, a portion of the tumor was fixed with 2.5% glutaraldehyde for TEM analysis [36]. The remains of animals that were not recovered from the necropsy or preserved in paraformaldehyde, were placed in a yellow polyethylene bag for pathological residues, stored at 4 °C and transported to a collection center for biological-infectious hazardous residues for subsequent incineration [37].

### In silico analysis

In silico analyses with the molecular structures of GA and Myr were performed with PubChem (https://pubchem.ncbi.nlm.nih.gov/) and ACD/I-Labs© (https://ilab.acdlabs.com/iLab2/), to predict their pharmacological activities. The identification of target pharmacophores was carried out with Zinc15 (http://zinc15.docking.org/) [38] and Similarity Ensemble Approach (SEA) model (http://sea.bkslab.org/) [39], to find proteins with binding sites for the active compounds through an inverse protein-ligand approach. The target potentials were selected based on *P-Value* or *Max TC* parameters, provided by the server.

### Statistical analysis

The results of this study are presented as the mean ± standard deviation (S.D.) of triplicates obtained from 2 to 3 independent assays. The statistical analysis was performed with one-way ANOVA for parametric data with normal distribution, and comparisons were made with normal/pathological controls through the posthoc test of Tukey-Kramer and Dunnett, in Minitab® software (version 16.1). The differences observed were considered significant when *p* ≤ 0.05.

## Results

### Biological activity of GA and Myr in cell lines

In this study, the biological activity of GA and Myr was evaluated against ovarian adenocarcinoma cells. GA reduced cell viability by 50% in SKOV-3 and OVCAR-3 cells at 50 and 43 μg/mL respectively, while Myr showed activity at 166 and 94 μg/mL in the same cell lines, compared to the treatment of negative control group (vehicle) (*p* ≤ 0.05, Dunnett) (Fig. 2a and b). Positive control with paclitaxel administered at 5 μg/mL diminished viability to 50% compared with the vehicle group (data not shown). These results demonstrated that OVCAR-3 cells are more sensitive to the effect of GA and Myr compared to SKOV-3 cells. In addition, the cytotoxic activity of both compounds was evaluated in the BEAS-2B transformed/non-tumorigenic cell line; GA and Myr showed activity at 25 and 64 μg/mL respectively, compared to the vehicle treated group (*p* ≤ 0.05, Dunnett) (Fig. 2a and b), demonstrating low selectivity in their activity. Recent studies have related the capacity of polyphenols to induce oxidative stress through the generation of reactive oxygen species (ROS) with their biological activity in cancer, since ROS can act as a second messenger and modulate the activity of various biologic processes related to the cell cytoskeleton, cell division, and cell death [14, 16]. Therefore, the production of intracellular ROS in SKOV-3 was determined during treatments with GA and Myr for 24 h. Both compounds increased the ROS production by 42 and 34% respectively, compared with the 3.5% observed in the vehicle group or 76.5% with 0.3% H_2_O_2_ group (*p* < 0.05, ANOVA) (Fig. 2c). Additionally, changes in the cell morphology were observed by appreciation in microscopy analyses during the administration of the treatments, such as cell rounding and individualization (GA, Myr, paclitaxel in PCM at 40X), cytoplasmatic reduction (Myr and paclitaxel in TEM at 1000X), condensation of nuclear chromatin (Myr B.1 and paclitaxel C.1 in TEM), increase in cytoplasmic vesicles (GA, Myr, paclitaxel in TEM at 1000X), presence of autophagic vesicles (GA A.1 in TEM), mitochondrial alterations (Myr and paclitaxel in TEM at 1000X), and absence of mitotic division in comparison with vehicle group that present chromosomal segregation (vehicle at 1000X and D.1 in TEM) (Fig. 2d). These changes suggested the activation of an apoptotic process. Thus, we proceeded to analyze this possibility by measuring cell death via the externalization of phosphatidylserine in the cellular membrane and alterations in the cell permeability. Treatments with GA and Myr for 24 h, induced apoptosis (18.9/8.1%) and necrosis (26.6/15.1%) in SKOV-3 cells respectively; this effect, although of less intensity, was similar to that observed with paclitaxel (*p* < 0.05, ANOVA) (Fig. 3a).

Recent studies have demonstrated that ROS are capable of inducing disorders in the network of microfilaments and microtubules of the cellular cytoskeleton, which participates in several important functions such as support, transport, traffic, and cell division [40]. GA and Myr generated changes in the structuration of actin and tubulin of SKOV-3 cells (Fig. 3b); in the case of actin, accumulation of polymerized actin and a decrease of membrane prolongations and filaments were observed; while in the case of tubulin, cumulus of stabilized tubulin and an increase in the number of microtubules were observed (Fig. 3b). These changes were also present in cells treated with paclitaxel, but absent in cells treated with the vehicle (Fig. 3b). Possibly, these changes correlate with the absence of cell division found in TEM results (Fig. 2d).

Finally, cells treated with GA increased the G_2_/M phase (8.3%), while in cells treated with Myr the G_0_/G_1_ phase increased (78%), in comparison with non-treated cells from the vehicle group (*p* < 0.05, ANOVA) or the increase observed in G_2_/M phase (15.3%) in the cells treated with paclitaxel (Fig. 3c). These results correlate with the increase in the ROS production and with the changes in the cell morphology of SKOV-3.

### Antineoplastic activity of GA and MYR in mice xenotransplanted with SKOV-3

Therefore, based on the previous results, studies were conducted in the animal model to corroborate the therapeutic effect of GA and Myr in ovarian cancer. The p.t. administration of GA and Myr (50 mg/kg/2 alternate days per week) for 4 weeks after tumors have developed did not induce behavioral changes (agitation, tremor, drowsiness, loss of appetite) or toxicity signs (dyspnoea, photophobia, blindness, diarrhoea, heart failure, muscle weakness, seizures, and epithelial pigmentation) in rodents in the first hours of the study. Additionally, changes in the body weight of rodents were monitored for 28 days. The group treated with GA presented a 7.4% decrease, while the Myr group obtained a 1.4% increase. However, the observed changes were not significant concerning the control group treated with vehicle (*p* > 0.05, Tukey), or paclitaxel (Fig. 4a). At the end of the treatments, rodents were euthanized to recover the tumor lesions for further macro- and microscopic morphology analysis. Although in all groups (treated or not), tumors with similar characteristics were found, such as an ovoid shape, a smooth surface, and presence of vasculature, changes in color were observed (Fig. 4b; Table 1). The tumors of the GA group showed a yellowish color, whereas those from the Myr group were rose-colored, while the paclitaxel-treated group developed whitish tumors and the vehicle group a more yellowish-colored tumors (Fig. 4b). However, significant differences in tumors’ weight were observed. The greater tumor mass was produced in those mice treated with vehicle (0.68 ± 0.16 g), followed by the group treated with Myr (0.11 ± 0.06 g), then by the GA group (0.078 ± 0.04 g), and finally those from the paclitaxel group (0.045 ± 0.01 g) (*p* > 0.05, Dunnett) (Table 1). These results correlate with the tumoral volume obtained in the different lesions. The GA, Myr and paclitaxel groups showed a significant decrease in tumor volume from 7 to 28 days after treatment, remaining at the end with the following volumes: 67.5 ± 11.6 mm^3^ with GA, 73.2 ± 15.3 mm^3^ with Myr, and 42.4 ± 18.6 mm^3^ with paclitaxel, in comparison with the tumor volume of the vehicle group (364.3 ± 28 mm^3^), confirming the inhibitory effect of the treatments (*p* ≤ 0.05, Dunnett) (Fig. 4b; Table 1). These results agree with those found with the larger diameter of tumoral lesions in the different groups, before and after the treatments. Mice treated with GA and Myr showed a stable progression of the disease, with a relative increase in size of 11.6 and 3.4% respectively, whereas in the paclitaxel group there was a 23.3% reduction, in comparison with the vehicle group that presented a significant increase of 47.4% (*p* ≤ 0.05, ANOVA) (Fig. 4c; Table 1). Finally, to perform an in-depth study of the antineoplastic activity of GA and Myr, imaging studies were carried out to corroborate the previously obtained results.

**Table 1:** Morphological characteristics of ovarian tumor lesions treated with GA and Myr

Treatments	Gallic acid	Myricetin	Paclitaxel	Vehicle
Weight (g)	0.078 ± 0.04*	0.11 ± 0.06*	0.045 ± 0.01*	0.68 ± 0.16
Larger diameter (mm)	6.0 ± 2.0*	5.8 ± 1.4*	4.6 ± 0.57*	11.6 ± 0.57
Tumor volume (mm^3^)	67.5 ± 11.6*	73.2 ± 15.3*	42.4 ± 18.6*	364.3 ± 28
Vascularity	–	–	–	+
Fibrosis	+	+	+	+
Morphology	Ovoid mixed	Loculated ovoid	Homogeneous ovoid	Loculated ovoid

Results show the mean ± S.D. of two biological replicates (*n* = 5)

*, *p* < 0.05 vs values of vehicle group (0.5% DMSO in 1X PBS, ANOVA)

(+), present; (−), absent

#### Imaging and histopathological studies of tumor lesions

**Fig. 5:**
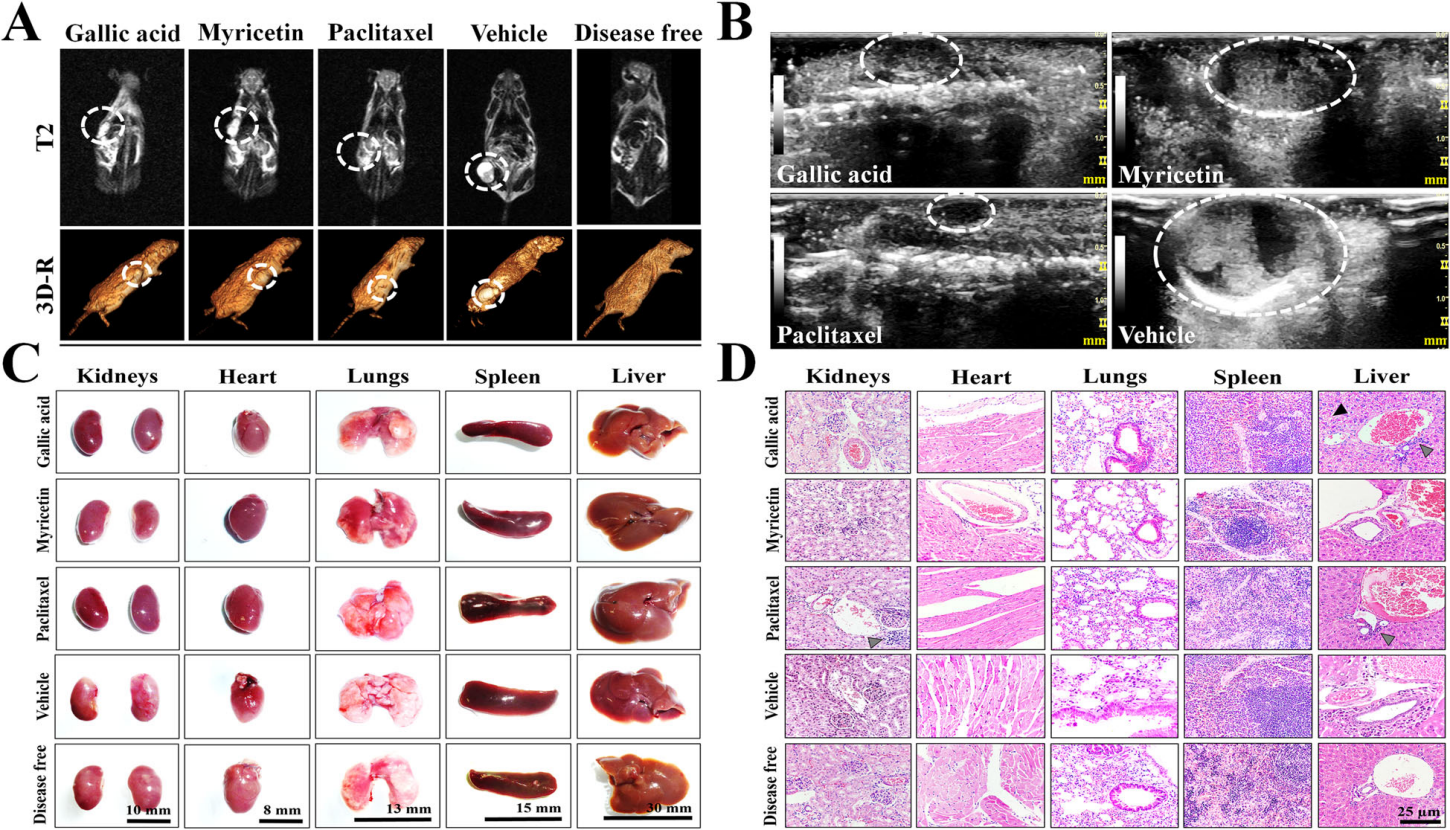
Imaging and histopathologic studies in mice treated with GA and Myr.NMR (**a**) and USG (**b**) were performed to observe densitometric and morphological changes in the tumoral lesions, as well to discard metastatic processes during treatments. The tumor lesions were delimited with a white circle in the corresponding images of both studies. Observations of the anatomical morphology (**c**) and the histological patterns (**d**) of organs extracted after laparotomy were made, to discard tissue lesions caused by treatments (50 mg/kg in 2 alternate days per week, 4 weeks, peritumoral route). Histopathology images were taken at 40X magnification and color arrows indicate loss of hepatic parenchyma (black arrowhead) or leukocyte infiltrates (grey arrowhead). Results are representative of two biological replicates (*n* = 5). NMR, nuclear magnetic resonance; T2, transverse relaxation times; 3D-R, 3D-reconstruction; USG, ultrasonography

Simple full-body resonances in supine decubitus with T1, T2, and STIR sequences were performed, as well as coronal reconstructions. The imaging studies showed ovoid tumors in all groups, located in the subcutaneous cellular tissue, which presented regular and well-defined borders, isointense with respect to the soft tissue (T1), and hyperintense/heterogeneous at expense of solid component and scarce liquid inside (T2 and STIR) (Fig. 5a; Figure S1). However, the vehicle group presented an abundant liquid component, possibly related to the pathology development (Fig. 5a; Figure S1). Finally, metastatic processes were absent in all treatments (Fig. 5a). The imaging study was complemented with USG in real-time and Doppler techniques (power and color modalities) to morphologically characterize the tumor lesions in mice. Treatments with GA, Myr, and paclitaxel presented similar characteristics, such as heterogeneous echotexture, with the predominance of solid component, diffuse areas related to fibrosis and absence of vascularity (Fig. 5b). While, the vehicle group presented predominance of a cystic component, internal septa, and central vascularity (Fig. 5b). Additionally, histological analysis of the lesions revealed a medullary neoplastic stroma of mixed composition, with a solid pattern in “comedo-type” for GA, Myr, and paclitaxel, presence of extensive central necrosis surrounded by leukocytic infiltrates, stromal retraction, fibrosis, decreased vascularization and induction of apoptosis (Fig. 4d). While, lesions treated with vehicle presented a desmoplastic and microcystic pattern, characterized by a broad band of fibrosis, adhesions, and loss of neoplastic stroma, as well as an increase in peritumoral vascularization, and acute to moderate chronic inflammation without residual organ, all of these characteristics that correspond to serous papillary carcinomas (Fig. 4d).

### Toxicological evaluation of treatments with GA and Myr

The anatomical observation of liver, heart, spleen, lungs, and kidneys was performed in search of morphological alterations. However, the morphology of the organs was similar in all groups compared with the organs of mice treated with vehicle or non-xenotransplanted mice (Fig. 5c). Additionally, morphometric analyses were performed to find differences in weight and diameter of the organs in each group. The GA group presented the largest spleen (0.2 ± 0.05 g/23.6 ± 4.7 mm), as well as the liver (2.1 ± 0.2 g/31.3 ± 2.1 mm); while the paclitaxel group showed the smallest lungs (0.18 ± 0.01 g/18.6 ± 0.6 mm) and spleen (0.18 ± 0.01 g/12.0 ± 1.5 mm); in both cases compared with the vehicle group (*p* ≤ 0.05, ANOVA) (Table 2). Subsequently, histological sections of the organs were performed in search of histopathological lesions. The characteristic stroma of each organ was observed with a 10X magnification (Figure S2). In the GA group, the loss of hepatic parenchyma and leukocytic infiltrate can be observed at 40X (Fig. 5d), whereas in the paclitaxel group, acute to moderate chronic inflammation was observed in liver and kidney at 40X (Fig. 5d). The morphological differences mentioned above were absent in the organs from Myr and vehicle groups, as well as in the organs of mice without pathology (Fig. 5d). Finally, the hematological and biochemical parameters were determined and compared concerning the vehicle group or the reference values ​​reported for mice. The GA and Myr groups had a light leukopenia of 3466.7 ± 1050.4 × 10^6^/mm^3^ and 3766.6 ± 1644.2 × 10^6^/mm^3^ (*p* ≤ 0.05, ANOVA) respectively (Table 3). In addition, the GA group presented neutrophilia (52.6%), hypertransaminasemia (ALT: 150.7 ± 25.60 U/L), and hypoazotemia (BUN: 13.3 ± 1.4 g/dL, urea: 33.4 ± 7.4 mg/dL, and creatinine: 0.41 ± 0.1 mg/dL), which could suggest the presence of a chronic hepatitis, liver failure and necrosis, which correlate with the loss of liver parenchyma, previously reported in the histopathological findings (*p* ≤ 0.05, ANOVA) (Table 3). While, the paclitaxel group showed signs of azotemia (urea: 63.7 ± 7.7 mg/dL and BUN: 30.3 ± 3.8 g/dL), which may indicate renal failure and correlation with the leukocytic infiltrate observed in the kidneys during the histological analysis (*p* ≤ 0.05, ANOVA) (Table 3). These results suggest that the GA administration induced a chronic toxicological effect, which may be related to the concentration of compound used in the study. However, the administration of Myr did not cause histopathological or biochemical alterations; therefore, Myr could be used as a alternative treatment for ovarian cancer.

**Table 2:**
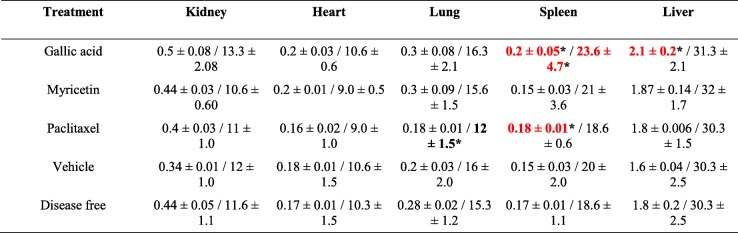
Morphometric analysis of organs extracted from *Nu/Nu* mice treated with GA and Myr

Results show the mean ± S.D. of two biological replicates (*n* = 5)

Measurement of weight (g) / larger diameter (mm) from each organ

The numbers in red and bold black indicate higher and lower differences respectively in comparison with values obtained with the vehicle group

*, *p* < 0.05 vs values of vehicle group (0.5% DMSO in 1X PBS, ANOVA)

**Table 3:**
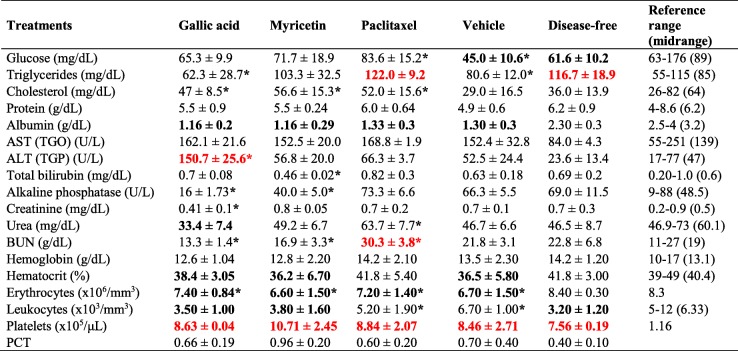
Paraclinical studies in *Nu/Nu* mice treated with GA and Myr

Results show the mean ± S.D. of two biological replicates (*n* = 5)

The numbers in red and bold black indicate higher and lower differences respectively in relation to reference values for mice

*, *p* < 0.05 vs values of vehicle group (0.5% DMSO in 1X PBS, ANOVA)

Reference range, minimum and maximum normal value for the analyte of interest in mice and the respective midrange [32, 41, 42]

*AST (TGO)* aspartate aminotransferase, *ALT (TGO)* alanine aminotransferase, *BUN* blood urea nitrogen, *PCT* platelecrit

### Pharmacological properties and therapeutic targets of GA and Myr

In silico assays were conducted with the molecular structures of GA and Myr in ACD/I-Labs©, ZINC and SEA to determine the molecular mechanism, the pharmacological properties, the possible toxicological effects, as well as the doses and the therapeutic targets of these compounds. The results obtained suggest that GA could induce the activation of ATM/Chk2/p53 and the inhibition of the carbonic anhydrase IX, COX-2/NF-kB and GSH signaling pathways, through the ROS production [25] Also, Myr was found to be a general inhibitor of protein kinases, such as PI3K-PKB/Akt/m.TOR, MEK1, Fyn, and JAK1-STAT3, among others [22, 24]. GA and Myr had a moderate toxicological effect, as well as an LD_50_ / maximum recommended daily dose (MRDD) of 3300 / 18.48 mg/kg for GA, and 120 / 2.24 mg/kg for Myr, respectively (Table 4). Finally, a comparison was made between the structures of GA and Myr; this analysis showed that both compounds have three hydroxyl functional groups, linked to an aromatic benzene ring that allows them to interact with the ATP binding site of different proteins, which are listed in Table 4, S1 and S2. However, more in-depth studies are required to confirm this interaction.

**Table 4:**
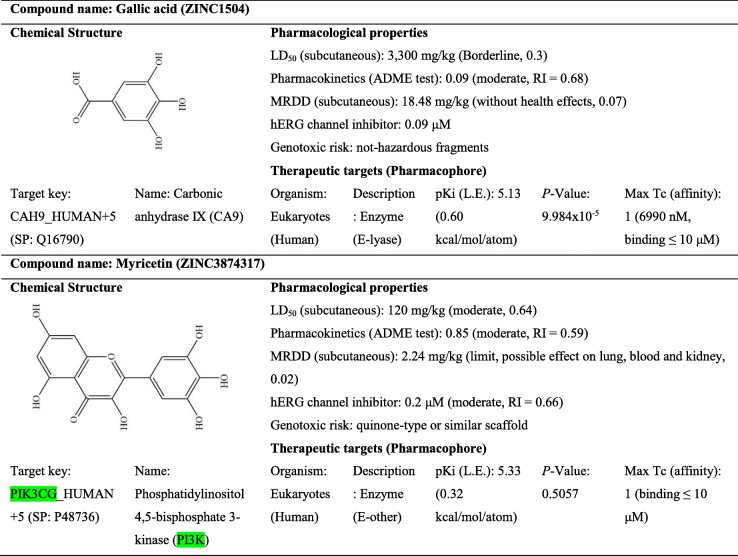
Pharmacological properties and therapeutic targets of GA and Myr

*LD*_*50*_ median lethal dose, *MRDD* maximum recommended daily dose, *ADME* absorption distribution metabolism and excretion, *hERG* human ether-a-go-go-related gene, *SP* Swiss-Prot protein sequence database (UniProt)

## Discussion

Results obtained in this study show that GA and Myr induced cell death, alterations in cell morphology, and cell cycle arrest in SKOV-3 cells at 50 and 166 μg/mL, respectively. These effects seem to be related to ROS production since GA and Myr increased the intracellular ROS in 42 and 34% in 24 h respectively. Recent studies have shown that the polyphenols are compounds with pro/anti-oxidant action in the cells, through the modulation of intracellular ROS and the induction of oxidative stress [16]. Additionally, studies have linked the ability of polyphenols to induce ROS with their biological activity in cancer, because ROS can act as second messengers and modulate the activity of different cellular processes [14, 16]. However, in-depth studies are necessary to demonstrate this cause-effect relationship in ovarian cancer. In addition, GA and Myr showed cytotoxic activity in BEAS-2B cells at 25 and 64 μg/mL respectively; this result suggests that, on the one hand, both compounds have a low selective effect in their therapeutic activity and, on the other hand, that the biological activity observed with both compounds is possibly related to the cellular phenotype. Therefore, the therapeutic activity of GA and Myr may be better tolerated by other normal-type cell lineages, but further studies are required to confirm this hypothesis.

The in silico analysis to determine the molecular mechanism and therapeutic targets of GA and Myr, revealed that GA might induce the activation of ATM/Chk2/p53 and the inhibition of COX-2/NF-kB and GSH [25], while Myr is a general inhibitor of protein kinases, such as PI3K-PKB/Akt/mTOR, MEK1, Fyn, and JAK1-STAT3, among others [22, 24]. Interestingly, both compounds could have an additional molecular mechanism of action based on results obtained in SEA approach. GA possibly can inhibit the carbonic anhydrase IX protein, which is a zinc-dependent metalloenzyme responsible for regulating the intracellular pH, through the conversion of CO_2_ and H_2_O into HCO_3_ by catalysis [43]; while Myr possibly can bind to tubulin and stabilize the microtubules in the cell cytoskeleton. None of these molecular mechanisms has been studied in depth. Although in this study we have proposed some target molecules only using the SEA approach, this analytical tool has been used widely to successfully predict the targets, toxicity and mechanism of action in diverse marketed drugs [44]; in addition, SEA has been proposed for the virtual detection and construction of a pharmacological network in the study of medicinal plants [45]. Whereby, the proposed interaction, GA-carbonic anhydrase IX or Myr-tubulin, most likely can take place in experimental and natural conditions, but additional studies will be required to confirm them. Recently, the fundamental role of carbonic anhydrase in different cancer types and parasitic pathologies was demonstrated [43]; similarly, other studies found that carbonic anhydrase is very abundant in ovarian cancer, unlike other types of cancer such as renal cancer [46]. On the other hand, PI3K/Akt/mTOR signaling pathway is dysregulated in diverse cancer types as glioblastoma or ovarian cancer, and mTOR is a key mediator of cellular processes such as growth, proliferation, metabolism, and angiogenesis [24]. Thus, the development of new drugs to inhibit these target proteins in cancer is an interesting perspective to address in the treatment of the disease. Moreover, diverse studies have demonstrated that the ovarian cancer is susceptible to the effect of several compounds that affect the cellular cytoskeleton, such as paclitaxel [47]. Therefore, these findings demonstrate that GA and Myr could be an interesting alternative for the treatment of ovarian cancer.

The in vivo assays with xenotransplanted mice with an ovarian cancer cell line, showed uneven growth of the tumor masses. Mice treated with GA and Myr by the p.t. route, showed a significant inhibition from the first week of administration, which reached the maximum value at the end of the fourth week, with 48.3 and 50% respectively, due to the induction of an apoptotic process. These results were similar to those obtained with the paclitaxel-treated group, which presented a 60.3% inhibition, while the vehicle group tripled the tumor size. Moreover, the histological analysis showed that all tumor lesions were high grade, which are associated with poor prognosis according to international guidelines [48]. To date, the main treatment for ovarian cancer with a degree of anaplasia IV is the administration of paclitaxel/carboplatin by the intravenous route, which has different action mechanisms [3]. Carboplatin can generate DNA adducts that inhibit cell proliferation [49], and paclitaxel can bind to the *β*-subunit of tubulin, thus stabilizing microtubules, blocking mitosis, and inducing cell death by apoptosis [50]. However, in the last stages of the disease, little effectiveness has been observed together with some toxicological effects [4, 5]. Therefore, the administration of GA and Myr by p.t. route can be considered as a viable and promising therapeutic procedure in the treatment of ovarian cancer and an option to replace or modify the traditional chemotherapy of this disease. Finally, the toxicological assay in rodents did not show behavioral changes or signs of toxicity during the p.t. administration of the different treatments. Myr did not induce changes in the morphometric, histopathological and paraclinical determinations in recovered organs or biochemical/hematological parameters concerning the vehicle group. However, GA induced hepatic necrosis and leukocytic infiltration, which was evidenced in the histological analysis. Likewise, hypertransaminasemia and hypoazotemia were observed, which are related to hepatic failure due to chronic inflammation caused by loss of liver parenchyma. Studies conducted to determinate sub-chronic toxicity of GA in F344 rats fed a diet containing 5% of the compound (*w/w*) for 13 weeks revealed a decrease in body weight and development of hemolytic anemia, hypertrophy of the centrilobular liver cells and changes in the proximal tubular epithelium of the kidney. Therefore, GA can be considered moderately toxic [51]. The results shown suggest a chronic toxicological effect during the p.t. administration of GA, which may be related to the concentration of the drug used in this study. However, Myr did not show histopathological or biochemical alterations and therefore, could be considered in the alternative treatment of ovarian cancer.

## Conclusions

GA and Myr presented biological activity against ovarian adenocarcinoma cells such as SKOV-3 (50 and 166 μg/mL) and OVCAR-3 (43 and 94 μg/mL) respectively, demonstrating differences of sensitivity in the effect of both compounds. Additionally, GA and Myr had cytotoxic activity in transformed/non-tumorigenic cell line as BEAS-2B (25 and 64 μg/mL), confirming low selectivity in their biological activity, possibly related to the cellular phenotype. Also, both polyphenol compounds induced morphological changes in SKOV-3 cells, mainly in the actin/tubulin cytoskeleton, cell cycle arrest and activation of cell death by apoptosis, through the generation of ROS. Finally, the peritumoral administration of GA and Myr (doses of 50 mg/kg) did not reveal behavioral changes or toxicity signs in rodents, but inhibited the development of ovarian tumor lesions, that allowed a stable progression of the disease. However, histological and paraclinical analysis of organs and blood extracted from mice during the toxicological study, revealed that GA induced hepatic necrosis, leukocyte infiltration, hypertransaminasemia, and hypoazotemia, which are related to hepatic failure due to chronic inflammation caused by loss of liver parenchyma; whereby additional studies are needed to find an adequate therapeutic dose for GA. In silico studies using the SEA approach allowed to suggest that carbonic anhydrase IX and PI3K proteins could be the most probable targets for GA and Myr respectively. Experimental and docking studies will allow to confirm this proposal. Therefore, GA and MYR could be considered as a starting point for the development of novel anticancer agents.

## Supplementary information

**Additional file 1.**

**Additional file 2.**

**Additional file 3.**

**Additional file 4.**

## Data Availability

All data generated or analyzed during this study are included in this published article (as well as supplementary information files). Raw data are available from the corresponding author on reasonable request.

## Abbreviations

ADME: Absorption distribution metabolism and excretion
ALT (TGO): Alanine aminotransferase
AST (TGO): Aspartate aminotransferase
ATM: Ataxia-telangiectasia mutated kinase
BUN: Blood urea nitrogen
Chk2: Checkpoint kinase 2
COX-2: Cyclooxygenase-2
DMSO: Dimethyl sulfoxide
FBS: Fetal bovine serum
Fyn: Proto-oncogene tyrosine-protein kinase
GA: 3,4,5-trihydroxybenzoic acid or gallic acid
GSH: Glutathione
hERG: Human ether-a-go-go-related gene
H&E: Hematoxylin and eosin
IC_50_: Half-maximal inhibitory concentration
i.p.: Intraperitoneal
JAK1: Janus kinase 1
LD_50_: Median lethal dose
MEK1: Mitogen-activated protein kinase 1
MRDD: Maximum recommended daily dose
mTOR: Mammalian target of rapamycin
MTT: 3-(4,5-dimethyl-2-thiazolyl)-2,5-diphenyl-2H-tetrazolium bromide
Myr: 3,5,7-trihydroxy-2-(3,4,5-trihydroxyphenyl)chromen-4-one or myricetin
NF-k*β*: Nuclear factor kappa *β*
NMR: Nuclear magnetic resonance
p53: Tumor suppressor protein
PBS: Phosphate buffer saline
PCM: Phase contrast microscopy
PCT: Platelecrit
PI: Propidium iodide
PI3K: Phosphatidylinositol 4,5-bisphosphate 3-kinase
PKB (Akt): Protein kinase B
p.t.: Peritumoral
ROS: Reactive oxygen species
SP: Swiss-prot protein sequence database (UniProt)
STAT3: Signal transducer and activator of transcription 3
STIR: Short-TI inversion recovery
T1/T2: Longitudinal/transverse relaxation times in coronal projections
TEM: Transmission electron microscopy
TOB: Toluidine blue
USG: Ultrasonography
