# Supplementary material for: Effect of Gallic acid and Myricetin on ovarian cancer models: a possible alternative antitumoral treatment
Source: BMC Complement Med Ther. 2020 Apr 10;20:110. doi: 10.1186/s12906-020-02900-z (PMC7149887; doi:10.1186/s12906-020-02900-z)
Supplement: Supplementary file 1 — Additional file 1. [file 12906_2020_2900_MOESM1_ESM.pptx]

## Slide 1
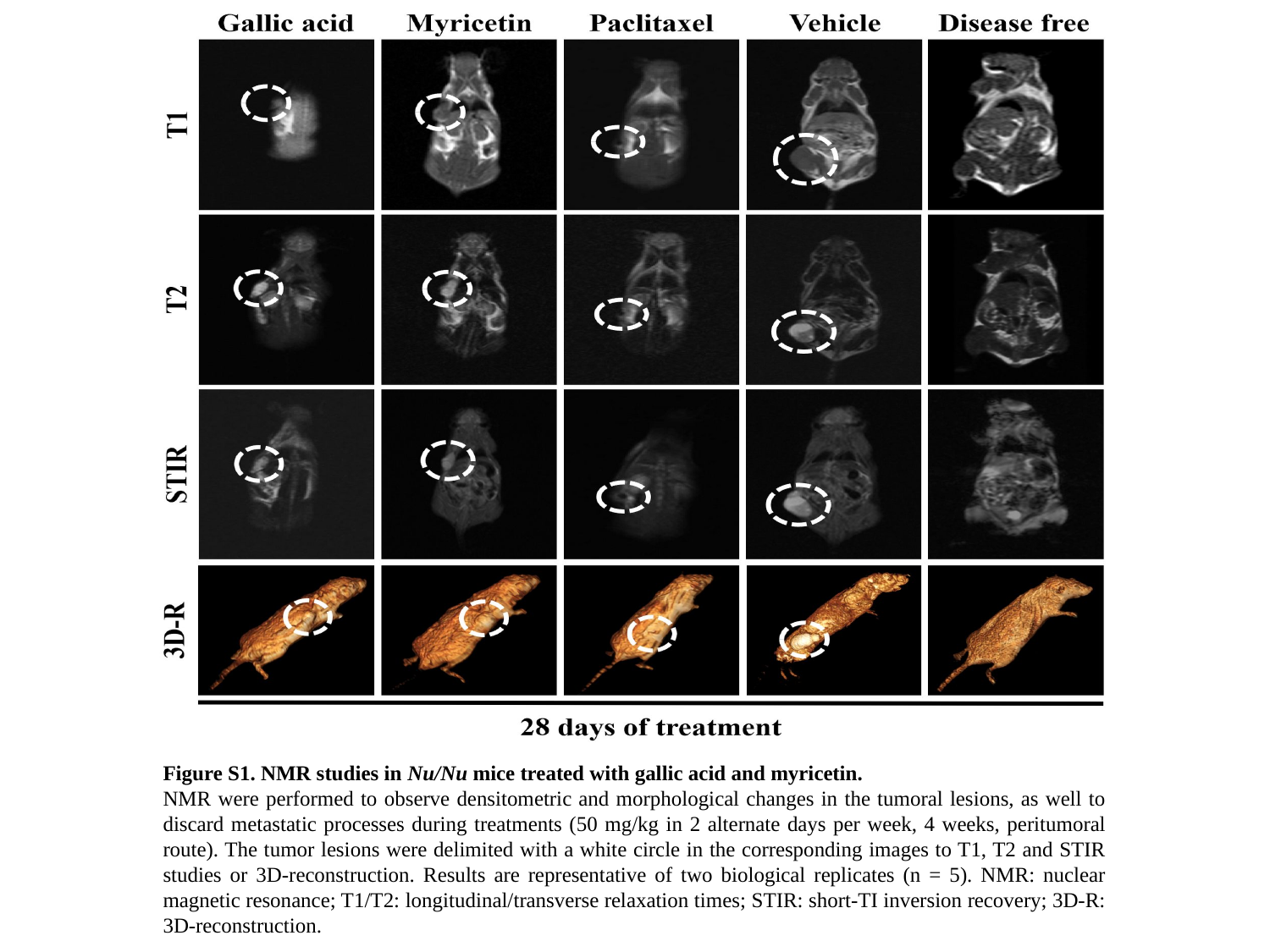

Figure S1. NMR studies in Nu/Nu mice treated with gallic acid and myricetin.
NMR were performed to observe densitometric and morphological changes in the tumoral lesions, as well to discard metastatic processes during treatments (50 mg/kg in 2 alternate days per week, 4 weeks, peritumoral route). The tumor lesions were delimited with a white circle in the corresponding images to T1, T2 and STIR studies or 3D-reconstruction. Results are representative of two biological replicates (n = 5). NMR: nuclear magnetic resonance; T1/T2: longitudinal/transverse relaxation times; STIR: short-TI inversion recovery; 3D-R: 3D-reconstruction.
