# Supplementary material for: Effect of Gallic acid and Myricetin on ovarian cancer models: a possible alternative antitumoral treatment
Source: BMC Complement Med Ther. 2020 Apr 10;20:110. doi: 10.1186/s12906-020-02900-z (PMC7149887; doi:10.1186/s12906-020-02900-z)
Supplement: Supplementary file 2 — Additional file 2. [file 12906_2020_2900_MOESM2_ESM.pptx]

## Slide 1
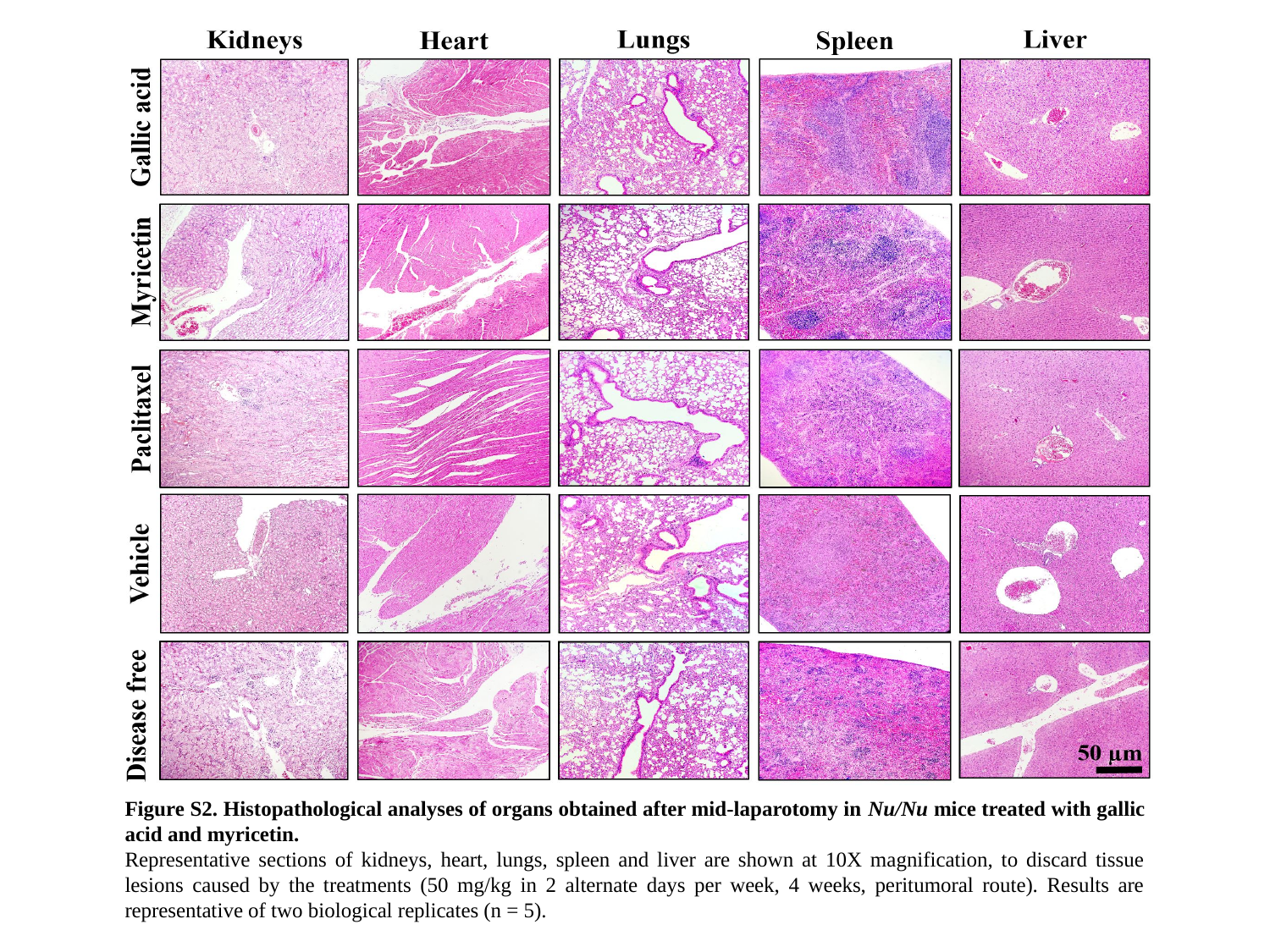

Figure S2. Histopathological analyses of organs obtained after mid-laparotomy in Nu/Nu mice treated with gallic acid and myricetin.
Representative sections of kidneys, heart, lungs, spleen and liver are shown at 10X magnification, to discard tissue lesions caused by the treatments (50 mg/kg in 2 alternate days per week, 4 weeks, peritumoral route). Results are representative of two biological replicates (n = 5).
